# Simulation-based User-centered Design: An Approach to Device Development during COVID-19

**DOI:** 10.1097/pq9.0000000000000427

**Published:** 2021-07-28

**Authors:** Nora Colman, Christopher Saldana, Kentez Craig, Nicole Edwards, Jennifer McGough, Carrie Mason, Kiran B. Hebbar

**Affiliations:** From the * Department of Pediatrics, Division of Pediatric Critical Care, Children’s Healthcare of Atlanta, Atlanta, Ga.; † George W. Woodruff School of Mechanical Engineering, Georgia Institute of Technology, Atlanta, Ga.

## Abstract

**Introduction:**

Since the onset of COVID-19, intubations have become very high risk for clinical teams. Barrier devices during endotracheal intubation protect clinicians from the aerosols generated. Simulation-based user-centered design (UCD) was an iterative design process used to develop a pediatric intubation aerosol containment system (IACS). Simulation was anchored in human factor engineering and UCD to better understand clinicians’ complex interaction with the IACS device, elicit user wants and needs, identify design inefficiencies, and unveil safety concerns.

**Methods:**

This study was a prospective observational study of a simulation-based investigation used to design a pediatric IACS rapidly. Debriefing and Failure Mode and Effect Analysis identified latent conditions related to 5 device prototypes. Design iterations made were based on feedback provided to the engineering team after each simulation.

**Results:**

Simulation identified 32 latent conditions, resulting in 5 iterations of the IACS prototype. The prototypes included an (1) intubation box; (2) IACS shield; (3) IACS frame with PVC pipes; (4) IACS plexiglass frame, and finally, (5) IACS frame without a plexiglass top.

**Conclusions:**

Integration of simulation with human factor ergonomics and UCD, in partnership with mechanical engineers, facilitated a novel context to design and redesign a pediatric IACS to meet user needs and address safety concerns.

## Abstract 2

Supplemental Digital Content is available in the text.

## INTRODUCTION

Since the onset of COVID-19, intubations have become very high risk for clinicians. Front-line teams face daily challenges as information evolves and clinical workflows and processes shift to address novel problems and changing needs. To achieve success under unexpected conditions, healthcare teams require a high level of adaptive capacity to adjust rapidly and respond to new information and challenges.^1,2^

To protect clinicians from COVID-19 aerosols, clinical teams must utilize aerosol containment devices during endotracheal intubation.^3–5^ Use of an enclosure device as an adjunct to PPE is supported by simulated studies that demonstrated reduced contamination of the intubator by containing aerosols inside of the box.^3–7^ The pediatric population varies in patient anatomy, bed sizes, and endotracheal tube (ETT) sizes, and poses unique challenges to a universal solution.

Human factor ergonomics (HFE), the study of how humans interact with machines, promotes the design of user-friendly devices to improve user performance, and reduce human errors.^8^ User-centered design (UCD) is a human factor technique that evaluates users and their interaction with the design through prototype testing and function analysis. Although widely accepted in consumer product development, observing a working prototype in a clinical context is problematic, given the difficulty in engaging busy clinicians and the inability to test the prototype on real patients.^9,10^ Inability to interface with the device in a clinical context and lack of attention to HFE leads to unsafe design flaws and obstacles that hinder the clinician’s ability to perform efficient work and deliver safe care.^11,12^

Simulation-based UCD is a patient safety improvement tool^13^ uniquely poised at the interface of HFE and UCD. Inefficiencies in the design are identified, and solutions are made to meet end-user needs and minimize risk to patients related to design flaws.^14,15^ This approach, which includes simulation, SAFEE debriefing, and Failure Mode and Effect Analysis (FMEA), is applied in systems, process testing, and architectural design development to identify and remediate latent conditions.^14,16,17^

Although adult intubation aerosol containment systems (IACSs) were being developed, there was a concern that it would not safely accommodate pediatric patients’ needs. Due to the time-sensitive nature of the pandemic, this process facilitated a user-centered, iterative approach for rapid development, testing, and evaluation of IACS prototypes.^4^

We describe how simulation anchored in HFE and UCD was applied to understand clinicians’ complex interactions with the IACS device, elicit user needs, identify design inefficiencies, and unveil safety concerns.

## METHODS

This study was a prospective observational study of a simulation-based investigation used to design a pediatric IACS rapidly. Simulation-based UCD occurred over 2 weeks, from March 16 to 27, 2020. Five-device prototype iterations were made, each evaluated with a single simulation, debriefing, and FMEA. The intubation process was repeated so that each clinician interacted with the device as the intubating physician, primary RT, and nurse. After each simulation, feedback was provided to the mechanical engineering team, a design iteration was made, and the modified prototype was re-tested using the same simulated scenario with the same degree of fidelity until thematic saturation was achieved and the final prototype was developed. This study was determined to be nonhuman subjects research by our institution’s Institutional Review Board.

### Conceptual Framework

#### Human Factor Ergonomics

HFE and UCD apply theories and methods to design user-friendly interfaces (tools/technology) to improve performance and understand factors that contribute to human error and unsafe care.^8,9,11^ UCD consists of 4 phases: understanding the context of use, specifying user requirements, evaluating the product, and designing solutions. In iterative design cycles, device evaluation informs goals for the next iteration.^8,18^ In the redesign, solutions are made to address unmet needs and remedy performance problems to eliminate hazards.^10,19,20^ The Systems Engineering Initiative for Patient Safety (SEIPS 2.0) model is anchored in HFE approaches and characterizes 5 work system elements: person, organization, technologies and tools, tasks, and environment.^12,17,21^ This framework illustrates the impact of system interactions and how the work system affects care processes and outcomes.^11,12,21^

#### Device Development

Before the simulation, we developed and implemented a COVID-19 intubation guideline that deviated significantly from our “usual” practice (**Appendix A, Supplemental Digital Content 1**, which displays special considerations for intubating covid-19 patients, http://links.lww.com/PQ9/A272). Our team learned and practiced the new process through simulation-based education, and the process was refined based on lessons learned from actual care delivery. Refresher trainings were required to maintain competency and prevent drift from newly implemented practices.^22^ During this time, ICU physicians informally collaborated with engineers to describe the context of device use. The device would be used for suspected and confirmed, non-emergent intubations of COVID-19 patients. Patients would be intubated by video laryngoscopy and the IACS had to accommodate varying patient sizes from infancy to adulthood. The first prototype tested was an IACS similar to that used for adults.

#### Identification of Testing Objectives and Scenario Development

Simulation-based UCD focused on prototype evaluation and devising solutions. Testing objectives were based on HFE principles that aimed to reduce environmental hazards, reduce risk of injury, provide safe delivery of care, optimize visibility, eliminate infection sources, and optimize physical and cognitive ergonomics (Table 1).^10,11^ A facilitator-guide anchored each step in the clinical scenario to human factor principles. Multiple design elements were evaluated as the facilitator prompted clinicians to interact with design elements under evaluation.^23,24^ This ensured that a wide range of latent conditions were identified. For example, clinicians were instructed to exchange the ETT for a smaller size after the first intubation attempt. This change required passage of supplies within the IACS, prompting the team to evaluate if the design minimized obstruction in the path of movement of supplies.

**Table 1.** Simulation Scenario Summary

Testing Objectives Reduce environmental hazards: The devise should minimize any hazards to staff or the patient during set up, use, and breakdown Reduce risk of injury: The design should minimize any obstruction in the path of movement, or any risk associated with any movement or repositioning of equipment, supplies, personnel, or the patient Provide safe delivery of care: The design should minimize environmental obstacles that may interfere with care delivery Optimize visibility: Design should facilitate optimal visualization to the patient Control or eliminate sources of infection: The design should minimize healthcare-associated infections or disease transmission Optimize cognitive ergonomics: The design should reduce decision making, minimize cognitive load, and enhance decision support Optimize physical ergonomics: The design should limit heavy lifting, repetitive movements, and physical exertion		
Scenario Progression	Intubation-related Tasks	Testing Objective
Phase 1: Team huddles before intubation Patient is on enhanced precautions receiving high flow nasal cannula at 10 L 100%	Team huddles outside of the room and assigns roles: Reviews airway plan Reviews medication plan: Atropine, Rocuronium, Ketamine Team dons PPE before entering room	
Phase 2: Team prepares pediatric IACS	RT lays all equipment, including airway adjuncts, on bed RT ensures that the Ballard is in-line with ventilator tubing Nurse attaches extension tubing for medication administration Physician positions patient appropriately	Reduce environmental hazards minimizing any risk of injury to staff or patient during set up Reduce risk of injury to minimize any obstruction during maneuvering of supplies Optimize visibility to the patient Optimize physical ergonomics by limiting heavy lifting or physical exertion during set up
Phase 3: Team prepares for intubation Patient is hypoxic and requires bag/mask ventilation	Patient is premedicated with Atropine RT turns off nasal cannula flow and removes noninvasive device (no apneic oxygenation) Physician and RT provide 2-person mask with V-E technique Nurse sedates patient with Ketamine and Rocuronium RT and physician provide bag/mask ventilation	Control and eliminate sources of infection to minimize exposure to aerosol generation Reduce risk of injury by minimizing obstruction of supplies and equipment while providing bag/mask ventilation Optimize visibility to the patient Provide safe delivery of care by minimizing any obstacles that would impact delivery of ventilatory support or medication administration Optimize physical ergonomics to limit repetitive movements Optimize cognitive ergonomics to minimize cognitive load
Phase 4: Intubation ETT is too large, and team must downsize ETT	Physician attempts to intubate with video laryngoscope and is cued that ETT does not fit RT and Physician exchange ETT for smaller size Physician intubates successfully RT removes stylet and inflates the cuff on the ETT RT checks for color change before auscultation RT and physician provide bag/mask ventilation RT attaches ETT to Ballard/vent circuit RT turns on ventilator and then tapes ETT	Reduce risk of injury by minimizing obstruction of supplies and equipment while providing bag/mask ventilation. Minimize any risk related to maneuvering of equipment, supplies, personnel, or patient Optimize visibility to the patient and video laryngoscope Provide safe delivery of care by minimizing obstacles that would impact intubation technique Optimize cognitive ergonomics to minimize cognitive load Control and eliminate sources of infection to minimize exposure to aerosol generation Reduce environmental hazards minimizing any risk of injury to staff during break down

RT, respiratory therapist.

#### Debriefing

Facilitated focused-debriefing identified latent conditions and potential active failures related to the design. Latent conditions were any weakness or deficiency in the design, such as limitations in accessibility, usability, and device safety. An active failure was defined as a potential error related to the latent condition, such as an unsafe practice, delay in care, or a procedural violation.^25^ The SAFEE (Summarize, Anchor, Facilitate, Explore, Elicit) debriefing approach identified error-provoking design elements defined by HFE principles.^23^ For example, the facilitator directed question: “how did visibility during direct laryngoscopy impact decision making?” explored the impact of design on cognitive ergonomics.

#### Failure Mode and Effect Analysis

FMEA is a proactive risk assessment tool used to seek out active and latent weaknesses in systems or processes and devise resolutions to remediate flaws.^26–28^ The FMEA scoring tool used at our institution to evaluate healthcare design and clinical systems,^28,29^ applied a 5-point Likert scale with seven categories. Each category was anchored to a specific description based on our institution’s safety event definitions, design FMEA, employee leave policy, and patient/family grievance policy (Table 2).

**Table 2.** FMEA Scoring Rubric

	5	4	3	2	1
Severity	Catastrophic	Major	Moderate	Minor	No Harm
	Patient Safety	Patient Safety	Patient Safety	Patient Safety	Patient Safety
	- Failure mode could result in permanent patient harm or death	- Failure mode could result in initial or prolonged hospitalization and cause temporary patient harm	- Failure mode could result in the need for increased patient monitoring, treatment, and/or in intervention, but there is no patient harm	- Failure mode reached patient but caused no harm	- Failure mode did not reach the patient
	Staff Safety	Staff Safety	Staff Safety	Staff Safety	Staff Safety
	- Failure mode results in loss of work for older than 90 d (long-term disability)	- Failure mode results in loss of work for older than 8 d up to 90 d (short-term disability)	- Failure mode results in loss of work for less than 8 d	- Failure mode results in missed work time during the same shift	- Failure mode did not result in any missed work
	Policies and Procedures	Policies and Procedures	Policies and Procedures	Policies and Procedures	Policies and Procedures
	- No policy/procedure is in place	- Policy/procedure is in place but needs to be modified	- Policy/procedure is in place but was not followed	- Policy/procedure is in place, but staff is unaware	- Policy/procedure is in place
	- Workflow does not support safe patient care	- Workarounds compromise clinical activities	- Workarounds created to optimize workflow do not align with best practices	- Workarounds have been created to optimize workflow	- Workflow supports safe patient care
	Equipment/Supplies/Technology	Equipment/Supplies/Technology	Equipment/Supplies/Technology	Equipment/Supplies/Technology	Equipment/Supplies/Technology
	- Staff experiences lack of functionality	- Staff experiences a reduction in performance and productivity	- Staff experiences a reduction in convenience	- Staff experiences annoyance	- Failure mode goes unnoticed by staff
	- Item completely fails to meet intended needs and performance is completely lost	- Failure can be overcome with modification, but there is some performance loss	- Failure can be overcome with modification, but there is no performance loss	- Failure can be overcome without modification or performance loss	- No need for modification, no loss in performance
	- Equipment/supplies/technology not available at all	- Equipment/supplies/technology available but does not function at all/retrieval delays care	- Equipment/supplies/technology available but does not function as intended or location is inconvenient	- Equipment/supplies/technology available but staff does not know how to use or access it	- Equipment/supplies/technology available and staff know how to use it
	Patient and Family Experience	Patient and Family Experience	Patient and Family Experience	Patient and Family Experience	Patient and Family Experience
	- Any grievance that requires referral to CMS	- Any grievance that requires involvement of Patient Rep or review by Patient Safety and Risk Management	- Any complaint or issue expressed to management that can be resolved promptly	- Any complaint or issue expressed verbally to staff that can be resolved promptly	- No complaints or issues expressed
	Cost to the Organization	Cost to the Organization	Cost to the Organization	Cost to the Organization	Cost to the Organization
	- Cost: ≥$250,000	- Cost: $100,000–$250,000	- Cost: $10,000–$100,000	- Cost: <$10,000	- Cost: None
	Regulatory Risk	Regulatory Risk	Regulatory Risk	Regulatory Risk	Regulatory Risk
	- Immediate jeopardy	- Conditional finding	- Standard finding	- Failure mode present. No violation	- None
Occurrence	Frequent	Often	Sometimes	Occasionally	Seldom
	Likely to occur more than once in a 24-h period	Probably will occur once a day	Possible to occur weekly	Possible to occur monthly	Unlikely to occur in a 6-mo period

Criticality score is calculated by multiplying Severity × Occurrence. Low priority (1–6), medium priority (7–14), high priority (15–19), and very high priority (20–25).

#### Setting

The Pediatric Intensive Care Unit at Children’s Healthcare of Atlanta is a 36-bed, high acuity, tertiary referral center that averages 200 intubations per year. Simulations took place in an in situ PICU patient room using a high-fidelity human toddler mannequin with intubation capabilities Gaumard PediHal S3004 (1 year old) (Guamard, Miami, Fla.). Two ICU physicians with extensive experience in the simulation-based process and architectural design testing delivered all simulations and facilitated debriefing and FMEA scoring. End users who participated in the simulation included attendings, fellows, nurses, and respiratory therapists; they used the IACS during actual patient care. Due to social distancing restrictions, simulations only involved staff at the hospital for patient care.

#### Simulation-based UCD

Each simulation began with a prebriefing to review testing objectives, orient end users to the mannequin and equipment, and review the COVID-19 intubation process. The simulation included a single scenario lasting 20–35 minutes. We used a detailed script with triggers during each simulation session to standardize the testing environment. Focused facilitation of the scenario prompted clinicians to interact with the IACS to elucidate the designs’ ergonomic impact (Table 1). A 25-minute debriefing immediately followed the completion of the scenario. Facilitators probed users to explore how the device impacted performance and safety, eliciting the impact of each latent condition identified.^23^

FMEA prioritized and categorized each latent condition identified.^16^ One of the facilitators scribed each latent condition and active failure identified during the debriefings into a preformatted FMEA template. An FMEA scoring session immediately followed each debriefing.

The facilitator summarized each latent condition discussed during the debriefing. End users assigned the latent condition to a severity category and determined the severity and occurrence score based on group consensus using the scoring rubric. This process was repeated for each latent condition identified. The facilitator remained impartial and did not influence scores. We calculated the criticality scores by multiplying severity and occurrence for each latent condition with equal weight given to each component. Of note, the occurrence score was defined as the projected frequency of COVID-19 intubations. It did not reflect the occurrence of staff exposure or transmission of disease. As the scoring team was not formally trained in FMEA and there was variation in the scoring team’s composition, the same simulationist facilitated all of the scoring sessions to maintain consistency.^14^ An FMEA report categorizing each threat was used by mechanical engineers to modify the design. Due to visitor restrictions, the engineers were not present at simulations.

## RESULTS

**Fig. 1.**
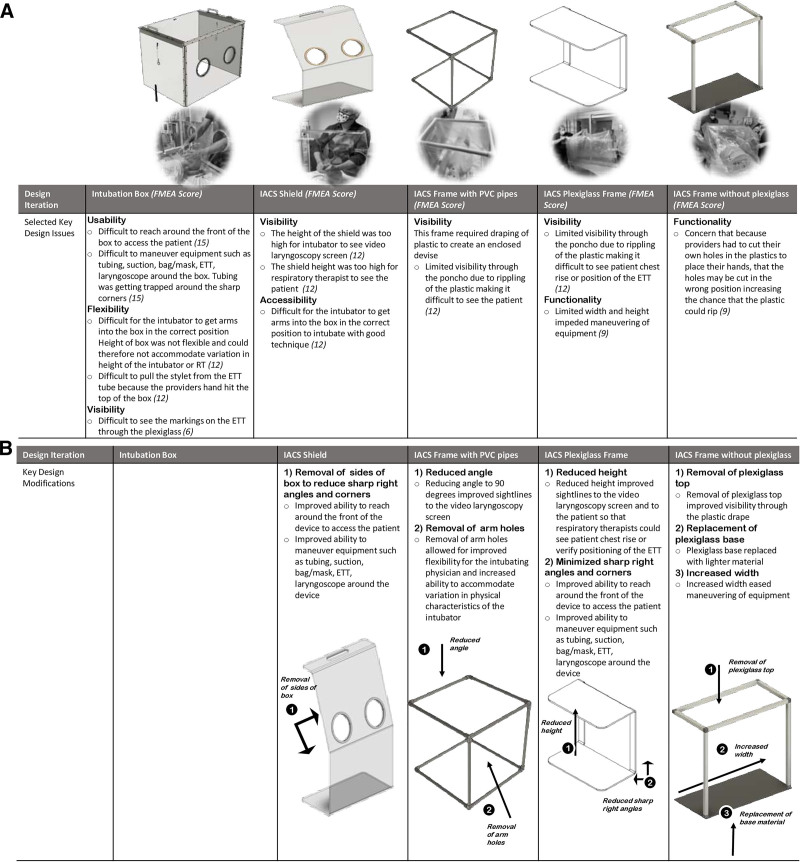
Pediatric IACS design iterations. A, Selected key design issues. B, Key design modifications.

Overall, 15 clinicians (nurses, physicians, and respiratory therapists) participated in the simulation, and 32 latent conditions were identified (Table 3). Based on simulation feedback, there were 5 iterations of the IACS prototype. The prototypes included an (1) intubation box; (2) IACS shield; (3) IACS frame with PVC pipes; (4) IACS plexiglass frame; and finally, (5) IACS frame without a plexiglass top (Fig. 1).

**Table 3.** Latent Conditions Identified during Simulation-based UCD

Latent Condition	Severity Category	Potential Active Failure	Severity	Occurrence	Criticality Score*
Intubation box					
- Concern that because the box was only opened on one side, the RTs had to reach around the front of the box to access the patient. It was difficult for the RT to reach around the box, thus requiring the intubator to hold the ETT in position while providing bag/mask ventilation	Patient safety	Lack of accessibility to the patient may result in loss of the airway, inability to oxygen/ventilate the patient. Ineffective bagging may lead to hypoventilation or hypoxia	5	3	15
- Concern that due to lack of flexibility of the plexiglass, tubing was getting trapped around the sharp corners and was kinking - Concern that suction, oxygen, or ventilator tubing may be too short to wrap around the box especially when using small ventilator circuits for infants	Patient safety	Kinking of tubing may cutoff suction or flow of oxygen to the bag/mask or ventilator circuit resulting in patient decompensation or even ETT dislodgement	5	3	15
- Concern that the width of the box was too wide and would be too big to fit on small cribs	Performance impact	Lack of flexibility to accommodate for varying pediatric bed sizes limits functionality of the device	5	3	15
- Concern that it was difficult for the intubator to get arms into the box in the correct position to intubate with good technique - Concern that the hand cut outs were not flexible enough to accommodate for variation in physical characteristics of the intubator	Patient safety	Poor positioning for the intubator may result in failed intubation attempts due to immobility and poor technique	4	3	12
- Concern that height of the box was not flexible and could therefore not accommodate for variation in height of the intubator or RT	Performance impact	Poor positioning for the intubator may result in failed intubation attempts due to immobility and poor technique	4	3	12
- Concern that it was difficult to anchor the box to the bed, especially if the head of the bed was elevated 30 degrees	Performance impact	This posed a risk to staff or the patient if the box was not adequately secured to the bed	4	3	12
- Due to the fixed height of the box, it was difficult to pull the stylet from the ETT tube because the provider’s hand hit the top of the box	Patient safety	Limited mobility inside the box may lead to accidental extubation or kinking of tubing	4	3	12
- Concern that because one side of the box was opened it was not possible to create a negative pressure	Staff safety	Lack of a closed system may expose staff to aerosols increasing the risk of pathogen exposure	3	3	9
- Concern that due to the large size of the box, it would be difficult to store	Performance impact	This may delay care if the device is not stored in an easily accessible location	3	3	9
- Concern that placing the box over the patient’s head may cause anxiety for the patient	Patient experience	This may result in a poor patient experience or increased anxiety requiring additional sedation	3	3	9
- Concern that because the box takes set up time, a patient may require initiation of respiratory support before putting the box in place	Staff safety	Inability to set up the box in a timely manner may mean that staff is exposed to aerosols if the patient requires bag/mask ventilation or emergent intubation	3	3	9
- Concern that the box was physically heavy and difficult to carry, maneuver, or position over the patient	Performance impact	This may lead to staff or patient injury	2	3	6
- Concern that the box takes up a significant amount of space on the bed, minimizing the space available for nurses and respiratory therapists to set up equipment	Performance impact	Limited space around the box necessitates an additional work surface space to be brought into the room so that equipment is easily accessed during intubation	2	3	6
- Concern that it was difficult to see the markings on the ETT through the plexiglass	Patient safety	This may result in the incorrect placement of the ETT which could impact ability to adequate ventilate/oxygenate the patient	2	3	6
IACS frame					
- Concern that it was difficult for the intubator to get arms into the box in the correct position to intubate with good technique	Performance impact	Poor positioning for the intubator may result in failed intubation attempts due to immobility and poor technique	4	3	12
- Concern that the height of the shield was too high for intubator to see the video laryngoscopy screen	Performance impact	Poor visualization to the video laryngoscopy screen may result in poor intubation technique and unsuccessful intubation attempts	4	3	12
- Concern that the shield height was too high for the RT to see the patient limiting the ability to see patient chest rise or verify positioning of the ETT	Patient safety	Inability to adequately visualize the patient may result in delay in care if changes in clinical status go unnoticed	4	3	12
- Concern that because one side of the box was opened it was not possible to create a negative pressure	Staff safety	Lack of a closed system may expose staff to aerosols increasing the risk of pathogen exposure	3	3	9
IACS PVC frame					
- Concern that there was limited visibility through the poncho due to rippling of the plastic making it difficult to see patient chest rise or position of the ETT	Patient safety	Inability to adequately visualize the patient may result in delay in care if changes in clinical status go unnoticed	4	3	12
- Concern that it was difficult to drape plastic around the frame and that set up was therefore time consuming	Performance impact	Concern that this may lead to a delay in patient care	3	3	9
- Concern that there was potential to rip the plastic during set up	Staff safety	Concern that ripped plastic may inadvertently result in contamination of staff	3	3	9
- Concern that because providers had to cut their own holes in the plastics to place their hands, that the holes may be cut in the wrong position increasing the change that the plastic would rip	Staff safety	Concern that ripped plastic may inadvertently result in contamination of staff	3	3	9
- Concern that because one side of the box was opened it was not possible to create a negative pressure	Staff safety	Lack of a closed system may expose staff to aerosols increasing the risk of pathogen exposure	3	3	9
IACS frame with plexiglass top					
- Concern that there was limited visibility through the plastic drape due to rippling of the plastic making it difficult to see patient chest rise	Patient safety	Inability to adequately visualize the patient may result in delay in care if changes in clinical status go unnoticed	4	3	12
- Concern that because providers had to cut their own holes in the plastics to place their hands, that the holes may be cut in the wrong position increasing the change that the plastic would rip	Staff safety	Concern that ripped plastic may inadvertently result in contamination of staff	3	3	9
- Concern that because the shield was an open system that there was not an ability to create a negative pressure space around the patient	Staff safety	Lack of a closed system may expose staff to aerosols increasing the risk of pathogen exposure	3	3	9
- Concern that the devise was not wide or tall enough to easily maneuver equipment inside the frame	Performance impact	Limited space may impact ability to provide necessary support	3	3	9
- Concern that it was difficult to drape plastic around the frame and that the setup was therefore time consuming	Performance impact	Concern that time spent setting up the device may delay patient care	3	3	9
- Concern that the device was physically heavy and difficult to carry, maneuver, or position over the patient	Performance impact	This may lead to staff or patient injury	2	3	6
IACS frame without plexiglass top					
- Concern that because providers had to cut their own holes in the plastics to place their hands, that the holes may be cut in the wrong position increasing the change that the plastic would rip	Staff safety	Concern that ripped plastic may inadvertently result in contamination of staff	3	3	9
- Concern that because the shield was an open system that there was not an ability to create a negative pressure space around the patient	Staff safety	Lack of a closed system may expose staff to aerosols increasing the risk of pathogen exposure	3	3	9
- Concern that the box was difficult to store due to large size	Performance impact	This may delay care if the device is not stored in an easily accessible location	2	3	6

RT, respiratory therapist. * Low priority (1–6), medium priority (7–14), high priority (15–19), and very high priority (20–25).

Latent conditions were categorized by performance impact, patient safety, staff safety, and patient experience. The design of the intubation box and IACS shield limited the ability to accommodate variation in provider physical characteristics (height, arm length, and hand size), resulting in poor positioning and suboptimal intubation technique. The fixed plexiglass box created obstacles that interfered with care delivery. Oxygen/suction tubing and ventilator circuit were unable to reach around sharp corners to the patient resulting in kinked tubing that impeded the delivery of oxygen/ventilatory support. The inability to create a negative pressure space increased the risk of user contamination during aerosol generation. The size and weight of the IACS increased the physical workload on users and made it difficult to store and maneuver the device. Visibility through the material and sightlines to the patient and video laryngoscope screen contributed to human error resulting in unsuccessful intubation attempts. The inability to see chest rise or the ETT position impacted awareness and decision-making, delaying the recognition of changes in patient clinical status.

The final IACS frame with plastic drape provided a degree of flexibility that the initial IACS box did not. In the final IACS prototype, the frame’s height and angle were adjusted to improve visibility to the patient and laryngoscope screen. The armholes were removed to provide flexibility and accommodate variation in provider characteristics. Reduced sharp right angles minimized kinking of tubing. Increased width minimized obstructions to the movement of supplies within the IACS. Replacement of the base with lighter material improved physical ergonomics related to its weight. The combination of plexiglass, plastic drape, provider goggles, and face shield generated significant glare. Removal of the top glass improved visibility by reducing the glare. The teams felt that the plastic drape provided enough protection against generated aerosols. The risk of unrecognized patient decompensation due to inadequate visibility outweighed the additional protection of the plexiglass top.

## DISCUSSION

During the COVID-19 pandemic, clinical care has become increasingly complex due to the constant evolution of information and changing protocols. There has been pressure to rapidly adapt new devices for intubation that potentially offer a higher protection level for healthcare workers. Integration of simulation with HFE, in partnership with mechanical engineers, facilitated a novel context to design and redesign a pediatric IACS prototype. Although centers have published the application of simulation in preparation and training in response to COVID-19,^1,30,31^ this is the first study to describe the integration of simulation with HFE in device development.

Inventing new solutions to novel problems in unprecedented conditions requires tremendous adaptive capacity, defined as the system’s ability to acclimate to complex, changing, and challenging conditions.^1,2^ Under high-stress conditions, clinical teams have a difficult time employing adaptative expertise.^2^ Upon introduction of a new intubation process, a practice which once felt routine and second nature, now felt complicated and foreign, adding cognitive load and intensifying stress. In the weeks following initial intubation training, skill retention proved to be challenging.^22^ Without a dissolution of COVID-19 in sight, development of the IACS created a long-term solution to reduce risk without dependence on human behavioral modification.^32^

Although studies have suggested that exposure to aerosols increase with the use of an intubation box,^33,34^ these reports focus on simulating particle generation or evaluating intubation technique in a silo, failing to completely represent the complexity of care delivery and the dynamic interactions of team members with each other and their work system during the task of intubation.^33,34^ We argue that hard wiring a new process in a high-stress and high stakes environment without the ability to prevent skill decay is prone to failure.

Simple adoption of an adult intubation box would have fast-tracked implementation. Yet, imagining integration in care and assuming “face validity” would have failed to identify prototype limitations and resulted in device failure or catastrophic outcomes. Demonstration of work as done revealed unintended consequences that the teams could not have imagined. Passing equipment such as suction tubing, removing the stylet from the ETT, repositioning the anesthesia bag so that the intubator could hold the ETT in place and bag simultaneously were complex tasks limited by design.

Simulation established a common ground that leveled perceptions regarding user needs across disciplines. Respiratory therapists assumed that physicians were ambidextrous when performing bag/mask ventilation. However, during simulations, each physician preferentially rotated the bag to ventilate with their right hand, highlighting an unrecognized provider preference. A shared understanding allowed for more effective positioning of equipment to support optimal technique. The resultant design of a frame instead of a box improved mobility of equipment and supplies within the IACS, optimizing the conditions under which clinicians coordinated tasks.

Although the number of suspected and confirmed COVID-19 patients requiring intubation has remained low in our PICU, the final prototype was integrated into practice. Before use, we ensured that the device fit on all crib and bed sizes. Initially, teams struggled to set up for intubation efficiently. As the IACS occupied significant space on the bed, inefficient setup resulted in inaccessible supplies. If teams did not have all supplies strategically laid out within the enclosure, they had difficulty maneuvering items in and out of the IACS. They noted that tape, ETTs, and airway adjuncts were catching on the plastic drape. In response, we developed a standardized supply checklist, training videos, and visual aids to guide the IACS setup. Latent conditions identified in the simulation were also noted in practice. IACS storage remained an issue due to its large size. Inability to secure the IACS to the bed precluded its use in patients who required ramping the bed for intubation. The time needed for set up precluded use in emergent intubations, and continued challenges with visibility to the patient precluded its use in known or presumed difficult airways. Use in practice highlighted the need for a mitigation plan for rapid device breakdown in the event of patient decompensation or cardiac arrest. This observation required additional process work and training. Contrary to concerns raised in simulation, patients tolerated positioning under the device without the need for additional anxiolysis. The simulation’s unintended benefit was that those who participated in device evaluation served as content experts and assisted clinical teams in identifying appropriate candidates and circumstances for IACS use, coached teams through set up, troubleshooting, and break down.

Work systems at individual institutions will differ in important ways in terms of user and environmental characteristics.^8^ The benefit of simulation is modifying the approach described here to accommodate variation in the clinical context, user groups, and institutional culture. Although the specific scenario described in this study and the findings identified may not be generalizable, the approach described can be adapted to evaluate other work system elements (new tool, technology, and task) across various clinical areas.

During the debriefing, anchoring HFE principles facilitated a discussion where users shifted away from thinking about systems of care and assessing the device through a lens that focused on the relationship between design and safety. Prioritized feedback in the form of FMEA provided the engineers with comprehensive feedback used to devise design solutions to address safety concerns with the highest risk as opposed to the tendency to make design modifications based on intuition.^9^

Simulation provides a platform to apply common usability testing principles to the healthcare system. This approach can enhance the safety evaluation of any new work system element before integration into care. Future research is necessary to validate this process for other devices over various clinical settings and contexts, explore its impact on safety, evaluate savings related to risk mitigation and cost avoidance associated with design retrofitting.

### Challenges and Limitations

This study has many limitations and challenges. The integration of simulation and iterative design has only been implemented at our center and is not a validated approach. The mechanical engineers who designed the IACS prototypes were not present at simulations due to social distancing restrictions. For the same reasons, the number of users who participated in simulation testing was also limited. We were unable to ensure that the same users were available to test all design iterations. Future considerations include video conferencing to integrate the engineers into the simulation. Simulations should also be conducted during scheduled blocked times to allow for undistracted participation and longer debriefing. Additionally, varying mannequin, bed, and crib sizes should be used to assess the device design fully.

## CONCLUDING SUMMARY

Simulation-based UCD highlights an unharnessed opportunity to increase the safety evaluation process during innovation adaptation and device development. Integrating simulation with HFE approaches facilitated the rapid development of an IACS to meet user needs and address safety concerns.

## DISCLOSURE

The authors have no financial interest to declare in relation to the content of this article.

## ACKNOWLEDGMENTS

We would like to acknowledge the Children’s Healthcare of Atlanta Simulation Center and the mechanical engineers at the Georgia Institute of Technology School of Mechanical Engineering for their assistance with this project.

## Supplementary Material


