# Supplementary material for: Simulation-based User-centered Design: An Approach to Device Development during COVID-19
Source: Pediatr Qual Saf. 2021 Jul 28;6(4):e427. doi: 10.1097/pq9.0000000000000427 (PMC8322510; doi:10.1097/pq9.0000000000000427)
Supplement: Supplementary file 1 [file pqs-6-e427-s001.pdf]

Special Considerations for Intubation

Patients on Enhanced Contact Droplet Precautions

Patients on Enhanced Contact Droplet Precautions may have high consequence infectious diseases. The process of performing aerosol-generating procedures presents an increased risk of exposure for health care professionals. This document is intended to serve as a guide and care should be customized to department specific workflows and patient population.

PREPARATION

1 Establish Team Plan

***Huddle outside of patient room and complete pre-intubation checklist**

Inside of the Room

5. Experienced intubation MD
6. Experienced intubation RT
7. Nurse: med. admin and documentation
8. Add members based on **clinical scenario and patient safety needs**

Outside of the Room

4. Nurses: Resource, Charge RN, documentation
5. Pharmacist or Med Nurse
6. Resource RT
7. Order Entry Provider
8. **Assign individual for emergency help**

2 Gather Procedural Items

Equipment

CMAC	LMA - oropharyngeal airway and nasopharyngeal airway
ETT securement device	
ETT	Large bore NGT
***HEPA filter**	***Disposable EtCO2 monitor**

Medications

- Atropine
- Rocuronium – *Consider 1.6 mg/kg to avoid chance of coughing*
- Ketamine 4 mg/kg
- Order continuous sedation

3 Don PPE

Enhanced Contact Droplet Precautions

1. Gown
2. Gloves
3. Goggles or Face Shield
4. N-95 mask with tight fitting seal

Other Considerations

1. Remove jewelry before entering room
2. Don/Doff utilizing buddy system
3. Double gloves and hairnet for intubating MD

INTUBATION

1 Pre-Procedure

Prepare for Rapid Sequence Intubation. *Note: Younger patients will likely require BVM.*

Elevate HOB to 45 degrees.

Place HEPA filter between mask and bag.

Pre-oxygenate with 100% O2 x 5 minutes with O2 delivery device currently in place. (Turn off prior to removing from patient to minimize aerosolization.)

2 Bag/Mask Tips

AVOID apneic oxygenation to maintain mask seal

Minimize bag/mask ventilation.

Utilize two-hand V-E grip.

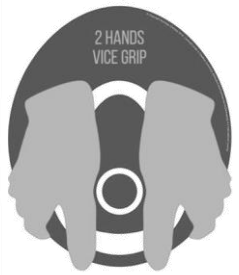

3 Procedural Method

Procedure

6. Intubate with video CMAC (no DL).
7. **Inflate cuff before BVM.**
8. Place HEPA filter and disposable EtCO2.
9. Confirm EtCO2 prior to auscultation.
10. Provide PPV.
11. **Clamp ETT.**
12. Disconnect bag and EtCO2.
13. Connect ventilator.
14. **Secure ETT after connecting to ventilator.**

POST-PROCEDURE

PPE Removal

Doff with an assigned co-worker observing the process. **The doffing process poses the highest risk for self-contamination.**

Refer to PPE Conservation and Reuse Guidelines for direction on item conservation.

Respiratory Care

Avoid unnecessary circuit disconnections.

Clamp ETT if disconnection is required.

OTHER CONSIDERATIONS

1. Consider **pre-assigning intubation MD and RT** each shift.
2. It can be difficult to hear verbal instructions and orders in full PPE. Speak clearly and **use closed-loop communication.**
3. Intubation may be challenging due to **risk of face shield fogging**.
4. **N-95 seals should be checked** prior to entering the room and adjusted as necessary.
5. Intubating MD should **double glove for intubation.** After intubation, remove one set of gloves and apply foam prior to doffing.

Appendix A. Special Considerations for Intubating COVID-19 Patients

(Colman et al) Simulation-based User Centered Design: An approach to device development during COVID-19
